# The capacity of HIV in the blood and the cerebrospinal fluid depending on antiretroviral drugs

**DOI:** 10.25122/jml-2021-0333

**Published:** 2022-05

**Authors:** Yuliia Igorivna Boiko, Vasyl Deoniziiovych Moskaliuk, Yurii Olexandrovich Randuk, Iryna Volodymyrivna Balaniuk, Ivanna Vasylivna Rudan, Tetiana Romanivna Kolotylo, Svitlana Romanivna Melenko

**Affiliations:** 1. Department of Infectious Diseases and Epidemiology, Bukovinian State Medical University, Chernivtsi, Ukraine

**Keywords:** human immunodeficiency virus infection, the load of HIV, blood, cerebrospinal fluid, antiretroviral therapy, acquired immunodeficiency syndrome, HIV – human immunodeficiency virus, CSF – cerebrospinal fluid, CNS – central nervous system, ART – antiretroviral therapy, RNA – Ribonucleic acid.

## Abstract

This study aimed to determine the capacity of HIV in the blood and cerebrospinal fluid of patients, depending on the reception of antiretroviral therapy (ART). Paired blood and cerebrospinal fluid samples were examined in 116 HIV-infected patients to determine the level of viral load in both biological fluids and the number of blood CD4+ lymphocytes. In patients receiving ART, the difference between the load of HIV in blood and cerebrospinal fluid (CSF) was significantly smaller than in untreated patients. Taking ART reduces the amount of HIV in the blood and CSF, but the dynamics of virus suppression in these biological fluids differ. The analysis revealed a statistically significant inverse relationship between the load of HIV in the blood and the number of CD4+ lymphocytes in untreated patients. There is a clear moderate positive correlation between the level of viremia and the clinical stage of HIV infection, as well as the duration of the disease. The number of CD4+ lymphocytes was expected to be inversely weakly correlated with the clinical stage of HIV infection and its duration. Accordingly, a direct correlation of mean strength was found between the levels of viral load in the blood and cerebrospinal fluid. There was a significant increase in the difference between the levels of HIV load in the blood and CSF compared with the average value in 25.6% of patients.

## INTRODUCTION

The issues of replication and concentration of human immunodeficiency virus (HIV) in various tissues and body fluids are still insufficiently studied. The solution to this problem is hampered by the lack of simple, cheap, and affordable methods for quantifying HIV in various tissue samples [1–3].

Despite the general pattern of lower HIV concentrations in the blood and lower virus load in body fluids after successful antiretroviral therapy (ART), there is evidence of conflicting results in determining the viral load in the blood and other biological samples of the same patient [1, 4–7].

The risk of autonomic virus replication in certain body tissues may be due to the insufficient penetration of antiretroviral drugs into various parts of the body. For example, the large size of the enfuvirtide molecule prevents it from penetrating the blood-brain and hematotesticular barriers [1]. The concentration of one of the non-nucleoside inhibitors, efavirenz, is only 0.5% in cerebrospinal fluid compared to plasma, although it reaches the required level of IC50 [4].

Studying the pathogenesis of the nervous system lesions after HIV infection, it became known that its direct effect lies in the cytopathogenic action directed at CD4+ cells of the nervous system: T-helpers, neuroglial cells, macrophages of the nervous system, vascular endothelial cells of the brain and spinal cord [5, 6]. In the early stages of HIV infection, the virus actively replicates in lymphoid tissue and microglia, so the presence of HIV in the cerebrospinal fluid (CSF) is assumed in the early stages after infection [2, 3, 5, 6, 9]. The viral load of cerebrospinal fluid and blood plasma is not always correlated: in some patients, the viral load of CSF far exceeds it in plasma and vice versa [7, 10–12]. The difference between the level of HIV in the serum and the CSF may reflect the formation of two independent reservoirs of HIV replication in the human body.

The purpose of this paper was to determine the capacity of HIV in the blood and cerebrospinal fluid of patients, depending on ART administration.

## MATERIAL AND METHODS

116 HIV-infected patients took part in the study. Patients were recruited using random sampling from individuals who visited the Chernivtsi Regional Centre for AIDS Prevention and Control. The research was carried out at this centre, as well as in the laboratory of the Ternopil Regional Council of the Regional Centre for AIDS Prevention and Control, the laboratory of the Ivano-Frankivsk Regional AIDS Center for Prevention and Control, and the Diagnostic Centre of Bukovina State Medical University.

All the patients were examined following the order of the Ministry of Health of Ukraine No. 551 dated 12.07.2010: “Clinical protocol for antiretroviral therapy of HIV infection in adults and adolescents” [8].

The main limiting factor for the inclusion of patients in the study was his/her consent to a spinal tap. The exclusion criteria were: age – less than 18 years and older than 60 years, traumatic brain injury and mental disorders before HIV infection, signs of organic disease of the central nervous system (CNS), current dependence on psychoactive substances, acute somatic diseases, and pregnancy.

The mean age of all patients was 34.5±7.4 years (range 18 to 60 years). In establishing the diagnosis, clinical and epidemiological data and the results of research methods such as serological and immunological (including determination of CD4+ lymphocytes) were considered. The level of CD4+ T-lymphocytes was examined after the disappearance of symptoms of concomitant acute infectious disease (at least 4 weeks).

Among the patients who participated in the study, the first clinical stage of HIV infection was detected in 5 patients (4.3%), the second stage – in 3 patients (2.6%), the third stage – in 12 patients (10.3%) and the fourth clinical stage – in 96 (82.8%) patients. 87 (75.0%) people did not receive ART before being included in the study.

The amount of HIV in the blood of patients (viral load) was determined in the laboratory of the Ivano-Frankivsk Regional Municipal Centre for AIDS Prevention and Control using test systems from the equipment manufactured by Hoffman La Roche. The Amplicor HIV-1 MONITOR Test used polymerase chain reaction (PCR) technology to detect very little genetic material (RNA) contained in human immunodeficiency viruses.

CSF selection was performed in sterile tubes, then aliquoted into micro tubes and stored in the frozen state. CSF studies were performed using the same method used for plasma because the chemical composition and rheological properties of CSF allow the use of this technique without further modification. The sensitivity of the method for blood plasma was 40 copies of RNA/ml, and the linear measurement ranged from 40 copies of RNA/ml (1.6 lg copies of RNA/ml) to 10 million copies of RNA/ml (7 lg copies of RNA/ml). Due to the inability to obtain a sufficient volume of CSF samples to perform studies with the same high sensitivity (the required analytical sample volume to obtain a result with a sensitivity of 40 copies of RNA/ml – 0.6 ml), the viral load in the cerebrospinal fluid was determined in a smaller sample volume (0.2 ml) with a sensitivity of 150 copies of RNA/ml (2.2 lg copies of RNA/ml) according to the instructions of the Abbott RealTime HIV-1 test system.

The number of CD4+lymphocytes in the blood was evaluated with flow cytofluorimetry using single-platform technology on a flow cytometer Becton Dickinson FACSCalibur using TriTEST CD3/CD4/CD45 reagent in TruCount tubes.

### Statistical analysis

We used descriptive statistics and the Shapiro-Wilk, Kolmogorov-Smirnov tests to verify the normal distribution of data. Spearman's rank correlation was used to identify a statistically significant relationship between phenomena, and the samples were compared using Fisher's exact test (if the number of values was less than 5). Nonparametric regression was used to assess the direction and dependence between phenomena. Finally, nonparametric one-way analysis of variance was used, the Mann-Whitney test for comparing two independent samples and the Kruskal-Wallis test for comparing average values in three or more independent samples.

## RESULTS

The paired blood and cerebrospinal fluid samples were taken from patients at each visit. A total of 124 paired blood samples and CSF were analyzed (Table 1). The number of CD4+ lymphocytes in the blood, the levels of HIV capacity in the blood plasma, and CSF were obtained for all 124 paired samples.

**Table 1:** The average values of the studied indicators in HIV-infected patients (according to the first visit).

Index	Average number* (95% CI)
**Number of CD4+ lymphocytes in the blood (cells/ml, n=116)**	112 (78–146)
**The level of HIV capacity in the blood (lg copies of RNA/ml, n=116)**	5.2 (5.0–5.4)
**The level of HIV load in the CSF (lg copies of RNA/ml, n=116)**	3.8 (3.6–4.1)

* – due to the lack of normal distribution of the obtained indicators, the truncated averages are given here and below.

The number of CD4+ lymphocytes in the blood ranged from 1 cell/ml to 846 cells/ml. Plasma loading in the blood ranged from indeterminate levels (below the analytical sensitivity of the test 1.60 lg copies of RNA/ml) to 6.82 lg copies of RNA/ml in cerebrospinal fluid – from indeterminate levels to 6.14 lg copies of RNA/ml. The level of HIV capacity in the cerebrospinal fluid varied in the same wide range as in the blood.

Among the examined patients, 18 patients received ART at the time of participation in the study; 87 patients had never taken them before. To study the effect of ART on the studied indicators, a comparative analysis of the content of CD4+ lymphocytes in the blood and the levels of HIV capacity in the blood plasma and CSF in the groups of patients with different therapy experiences was performed (Table 2). According to our observations, in patients receiving ART, the difference between the load of HIV in the blood plasma and CSF was significantly smaller than in those who did not take ART.

**Table 2:** Indicators of the content of CD4+ lymphocytes in the blood and the concentration of HIV in the blood and cerebrospinal fluid depending on antiretroviral drugs administration (cut average values from 95% CI, n=105).

Receiving ART	HIV concentration in the blood (lg RNA copies/ml)	Concentration of HIV in CSF (lg copies of RNA/ml)	The difference between the concentration of HIV in the blood and CSF (lg copies of RNA/ml)	The number of CD4+ lymphocytes in the blood (cells/μl)
**Never took ART (n=87)**	5.3 (5.1–5.5)*	3.8 (3.6–4.1)	1.5 (1.3–1.8) *	95 (56–134)
**Started ART (n=11)**	3.3 (2.6–4.0)*	3.1 (1.8–4.4)	0.4 (-0.2–0.9) *	155 (62–248)
**Long-term taking ART (n=7)**	1.5 (0.1–3.0)*	1.6 (0–3.2)**	-0.5 (-1.0–0.1)*	268 (152–384)**

* – statistically significant differences between all groups (P<0.05, Mann-Whitney test); ** – statistically significant differences between the group of patients who took ART for a long time and the groups of people who had never received ART and also started ART (P<0.05, Mann-Whitney test).

ART showed conflicting results in some patients on the background of long-term (more than 6 months) when the amount of virus in the cerebrospinal fluid exceeded its concentration in the blood (Table 1). In the group of long-term ART patients, signs of CNS damage were detected in 71.4% of cases (5/7). According to scientific publications, such patients are characterized by a slow decrease in the viral load of CSF when taking ART [2].

**Figure 1:**
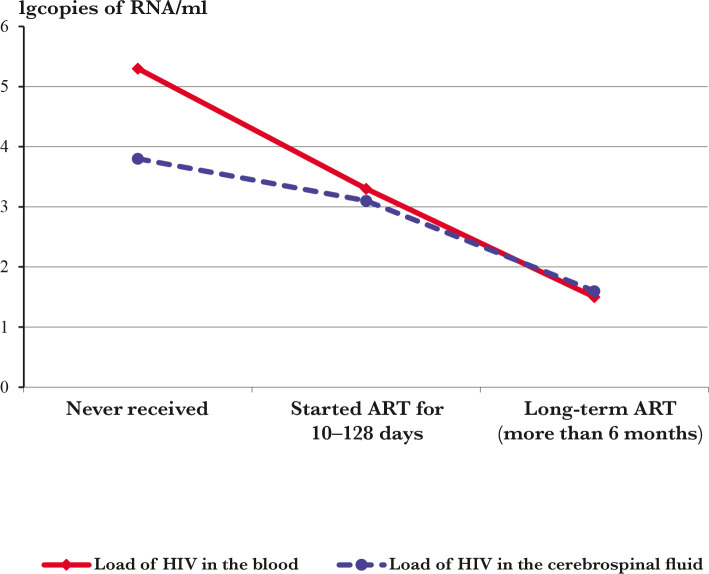
The average rates of HIV load in the blood and CSF in groups of patients with a different experience of ART.

The load of HIV in patients' blood and cerebrospinal fluid before and after receiving ART showed that ART reduces the amount of virus in both blood and cerebrospinal fluid, but the dynamics of virus suppression in these biological fluids differ significantly. The difference between the load of HIV in the blood and cerebrospinal fluid was significantly smaller in patients receiving ART than in untreated patients, reaching negative values in the group of patients with experience of taking drugs for more than 6 months. However, due to the insufficient number of patients examined prospectively before and after ART, it is impossible to establish an approximate timing of the onset of suppression of HIV replication in CSF (Table 2 and Figure 1).

As the use of antiretroviral drugs has a significant effect on the content of CD4+ lymphocytes in the blood and the level of viral load, both in the blood and in the cerebrospinal fluid, the results obtained in patients with no experience of ART are further used for statistical analysis (Table 3).

**Table 3:** Statistical dependence between the studied laboratory parameters and some clinical characteristics of HIV infection (the elements in the table indicate the values of the Spearman correlation coefficient, n=87).

Index	The load of HIV in the blood	The load of HIV in CSF	The number of CD4+ lymphocytes in the blood
**The load of HIV in the blood**	-	0.30*	-0.35*
**The load of HIV in CSF**	0.30*	-	0.14
**The number of CD4+ lymphocytes in the blood**	-0.35*	0.14	-
**Duration of the disease**	0.12	0.16	-0.48*
**Clinical stage of HIV infection**	0.24*	0.14	-0.51*

* – statistically significant relationship between indicators (P<0.05, nonparametric one-way analysis of variance).

The analysis revealed a statistically significant inverse relationship between the load of HIV in the blood and the number of CD4+ lymphocytes (r=-0.626–0.678, P<0.001). In addition, there is a clear moderate positive correlation between the level of viremia and the clinical stage of HIV infection (r=0.414–0.451, P<0.01), as well as the duration of the disease (r=0.391–0.430, P<0.01). The number of CD4+ lymphocytes was expected to be inversely weakly correlated with the clinical stage of HIV infection (r=-0.084–0.129, P<0.05) and its duration (r=-0.116–0.202, P<0.05). Accordingly, a direct correlation of mean strength (r=0.342, P<0.01) was found between the levels of viral load in the blood and cerebrospinal fluid.

In the group that did not receive ART (n=87), the viral load in the blood ranged from 2.6 to 6.9, averaging 5.3 lg copies of RNA/ml (95% CI 5.1–5.5); in CSF – from an indeterminate level to 5.9 lg copies of RNA/ml, on average – 3.8 lg copies of RNA/ml (95% CI 3.6–4.1, Table 2). The average viral load in the blood was higher than in the CSF, 1.5 lg copies of RNA/ml (P<0.05). The increase in viral load in the blood per 1 lg of RNA copies/ml corresponded to an increase in the load of HIV in the CSF by only 0.36 lg (nonparametric regression, P<0.05).

The predominance of HIV levels in the blood over the amount of HIV in the CSF was found in most patients – 89.7% (78/87). However, among these patients (in 25.6% (20/78)), there was a significant increase in the difference between the levels of HIV load in the blood and CSF compared with the average value: from 2.6 lg copies of RNA/ml and more, until the complete absence of virus in CSF against the background of a large amount of HIV in the blood (4.2 lg copies of RNA/ml). Among these patients, only one patient (5.0%) showed signs of CNS damage.

In the other 10.3% (9/87) of patients who did not receive ART, the number of viruses in the CSF was equal to or exceeded the viral load in the blood (taking into account the standard deviation of 0.25 lg obtained by the manufacturer when validating the test system and specified in instructions). Only one patient (5.0%) had signs of CNS damage among these patients.

## DISCUSSION

The results of this study showed that the capacity of HIV in CSF correlates with the concentration of HIV in the blood. However, the rather weak correlation force (r=0.342, P<0.01) and the significant both positive and negative differences between the amount of HIV in the blood and CSF in one-third of patients suggest that the degree of infection of CNS cells may be interrelated due to other factors, such as the concentration of certain cytokines in brain tissue and intracellular conditions of virus replication [3, 13–15]. Studying the causes of high concentrations of HIV in CNS tissue requires the search and analysis of other factors or substrates that can be either a catalyst or a consequence of active replication of HIV in the brain cells. It is possible that these substrates could be the subject of laboratory diagnosis and monitoring of HIV infection to optimize treatment and improve disease prognosis.

The viral load in the blood exceeded the load in the cerebrospinal fluid by 1.5 lg copies of RNA/ml in patients who did not receive ART. The higher concentrations of the virus in CSF and, accordingly, a shift in the difference between the amount of HIV in the blood and cerebrospinal fluid were observed to a lesser extent in the group of patients with signs of HIV-associated CNS damage (P<0.001). The results suggest that CNS dysfunction is associated with increased HIV replication in nerve tissue cells. However, it is impossible to establish the root cause of large amounts of virus in patients with signs of CNS dysfunction in a single study, as large numbers of viruses in CNS tissue can be the result of the destruction of the blood-brain barrier and high replicative activity and significant nerve cell infection.

The discrepancy between the levels of virus concentration in different tissues of a patient's body receiving ART seems quite natural [3, 16]. Against the background of insufficient adherence to therapy or selective adherence to drugs, the risk of autonomous active replication of HIV in different loci of the body increases. These features are most common in tissues that have a barrier to the free movement of drugs from the blood, such as the CNS (blood-brain barrier) and the genital tract (hematotesticular barrier) [17–19]. Thus, it is possible to create conditions for selective replication and selection of resistant variants of HIV in tissues where drug concentrations are reduced. Such reservoirs become a source of genetically different variants of the virus and disease progression, despite the apparent effectiveness of the treatment regimen, and promote microevolution of the virus by increasing resistance to the immune system and antiretroviral drugs [3, 20, 21].

## CONCLUSIONS

Taking ART reduces the amount of HIV in the blood and cerebrospinal fluid, but the dynamics of virus suppression in these biological fluids differ significantly. The difference between the load of HIV in the blood and cerebrospinal fluid was significantly smaller in patients receiving ART than in untreated patients, reaching negative values in the group of patients with a history of taking drugs for more than 6 months.

The load of HIV in the CSF of patients who did not receive ART correlates with the amount of HIV in the blood (r=0.342, P<0.01), and on average, it is lower than the blood level by 1.5 lg copies of RNA/ml. The increase in viral load in the blood per 1 lg of RNA copies/ml corresponded to an increase in the load of HIV in the CSF by only 0.36 lg (nonparametric regression, P<0.05).

We found a statistically significant inverse relationship between the load of HIV in the blood and the number of CD4+ lymphocytes (r=-0.6–0.678, P<0.001). In addition, there is a clear moderate positive correlation between the level of viremia and the clinical stage of HIV infection (r=0.414–0.451, P<0.01), as well as the duration of the disease (r=0.391–0.430, P<0.01). The number of CD4+ lymphocytes was inversely weakly correlated with the clinical stage of HIV infection (r=-0.084–0.129, P<0.05) and its duration (r=-0.116–0.202, P<0.05).
